# Genomic Epidemiology and Evolution of Duck Hepatitis A Virus

**DOI:** 10.3390/v13081592

**Published:** 2021-08-11

**Authors:** Enikő Fehér, Szilvia Jakab, Krisztina Bali, Eszter Kaszab, Borbála Nagy, Katalin Ihász, Ádám Bálint, Vilmos Palya, Krisztián Bányai

**Affiliations:** 1 Veterinary Medical Research Institute, Hungária krt 21, H-1143 Budapest, Hungary; feher.eniko@vmri.hu (E.F.); jakab.szilvia@vmri.hu (S.J.); bali.krisztina@vmri.hu (K.B.); kaszab.eszter@vmri.hu (E.K.); nagy.borbala@vmri.hu (B.N.); ihasz.katalin@vmri.hu (K.I.); 2 Veterinary Diagnostic Directorate, National Food Chain Safety Office, Tábornok utca 2, H-1143 Budapest, Hungary; BalintAd@nebih.gov.hu; 3 Ceva-Phylaxia Veterinary Biologicals Co., Ltd., H-1107 Budapest, Hungary; vilmos.palya@ceva.com; 4 Department of Pharmacology and Toxicology, University of Veterinary Medicine, István utca 2, H-1078 Budapest, Hungary

**Keywords:** duck hepatitis A virus, recombination, Hungary

## Abstract

Duck hepatitis A virus (DHAV), an avian picornavirus, causes high-mortality acute disease in ducklings. Among the three serotypes, DHAV-1 is globally distributed, whereas DHAV-2 and DHAV-3 serotypes are chiefly restricted to Southeast Asia. In this study, we analyzed the genomic evolution of DHAV-1 strains using extant GenBank records and genomic sequences of 10 DHAV-1 strains originating from a large disease outbreak in 2004–2005, in Hungary. Recombination analysis revealed intragenotype recombination within DHAV-1 as well as intergenotype recombination events involving DHAV-1 and DHAV-3 strains. The intergenotype recombination occurred in the VP0 region. Diversifying selection seems to act at sites of certain genomic regions. Calculations estimated slightly lower rates of evolution of DHAV-1 (mean rates for individual protein coding regions, 5.6286 × 10^−4^ to 1.1147 × 10^−3^ substitutions per site per year) compared to other picornaviruses. The observed evolutionary mechanisms indicate that whole-genome-based analysis of DHAV strains is needed to better understand the emergence of novel strains and their geographical dispersal.

## 1. Introduction

Infection caused by duck hepatitis virus 1 (DHV-1 or DHV type I), recently renamed as duck hepatitis A virus (DHAV), may be fatal in up to 95% of ducklings, typically under three weeks of age [1,2,3]. The disease is characterized by hepatic failures (enlargement, hemorrhages and necrosis), neurological signs (ataxia, opisthotonus) and the sudden death of affected birds [1,2,3,4]. The duck industry has been heavily affected by this virus; however, the disease can be efficiently prevented by vaccination [5].

DHAV belongs to the *Avihepatovirus* genus of the *Picornaviridae* family (https://talk.ictvonline.org/taxonomy/ accessed on 7 August 2021). The viral genome is a ~7.7-kilobase-long positive-sense single stranded RNA and consists of a single large open reading frame (ORF) flanked by the 5′ and 3′ non-coding regions. The polyprotein encoded by the single ORF is predicted to code for the VP0, VP3, and VP1 structural proteins and the 2A1, 2A2, 2B, 2C, 3A, 3B, 3C, and 3D non-structural proteins [6,7].

**Figure 1:**
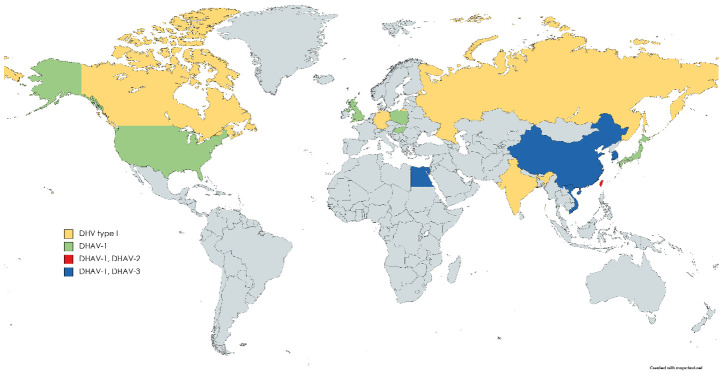
Geographic distribution of DHAV serotypes/genotypes. DHV type I is a historic assignment of DHAV introduced prior to the separation of serotypes.

Three serotypes/genotypes (DHAV-1, DHAV-2 and DHAV-3) of DHAV have been distinguished by serological and phylogenetic analyses. Outbreaks of DHAV-1 have been reported in many parts of the world, whereas DHAV-2 and DHAV-3 seemed to be geographically restricted to East and South Asia, although a DHAV-3 outbreak has recently been reported in Egypt (Figure 1) [1,2,6,7,8,9,10,11,12,13,14,15,16,17,18].

Outbreaks due to DHAV were frequently reported in Hungary until the early 1990s. Thereafter, perhaps due to the adequate control and prevention measures, occurrence of the disease was recorded only once, during an epizootic in 2004–2005 that affected 112 flocks of Pekin ducks and Muscovy ducks [18]. In the present study, we collected DHAV sequences from GenBank and from the archival sample that contained isolates from the 2004–2005 Hungarian disease outbreak, and performed molecular evolutionary analyses.

## 2. Materials and Methods

### 2.1. Viruses and Sequencing

All 10 Hungarian isolates available for the study originated from different duck farms in Bács-Kiskun County, Hungary. The carcasses were sent for duck hepatitis virus testing to the Veterinary Diagnostic Directorate, National Food Chain Safety Office, Budapest, Hungary. Liver samples were collected at autopsy and processed for virus isolation in embryonated Muscovy duck eggs as well as for diagnostic PCR.

For genome sequencing, the viral RNA was extracted from homogenized liver of the embryos using the DirectZol MiniPrep kit (Zymo Research, Irvine, CA, USA). For complete genome amplification, the viral RNA was reverse-transcribed with SuperScript^TM^ III Reverse Transcriptase (Thermo Fisher Scientific, Waltham, MA, USA) and specific reverse primers (Table 1). The mixture was incubated at 55 °C for 60 min and at 70 °C for 15 min. Three overlapping fragments of the genome were amplified in 25 µL PCR mixtures containing Phusion DNA Polymerase according to the manufacturers’ instructions and at 58 °C primer annealing temperature (Thermo Fisher Scientific, Waltham, MA, USA).

The PCR products were mixed for each isolate to obtain equimolar ratios for the three partially overlapping amplicons, and cDNA libraries were prepared for sequencing with an Ion Torrent PGM^TM^ or an Illumina NextSeq^TM^ 500 platform. Ion Torrent-compatible DNA libraries were prepared using the NEBNext^®^ Fast DNA Fragmentation & Library Prep Set for Ion Torrent (New England Biolabs, Ipswich, MA, USA) with the Ion Xpress^TM^ Barcode Adapters (Thermo Fisher Scientific, Waltham, MA, USA) according to the protocol recommended by the manufacturers. The emulsion PCR and subsequent template enrichment were performed with the Ion PGM^TM^ Template IA 500 Kit (Thermo Fisher Scientific, Waltham, MA, USA) and the Ion Torrent One Touch ES module, respectively. Sequencing was carried out on an Ion 316^TM^ Chip (Thermo Fisher Scientific, Waltham, MA, USA) using the Ion Torrent PGM^TM^. The cDNA libraries for Illumina sequencing were prepared by using the Nextera^®^ XT DNA Library Preparation Kit (Illumina, San Diego, CA, USA) and the Nextera^®^ XT Index Kit v2 Set A (Illumina, San Diego, CA, USA) according to the protocol of the manufacturer. The i5 and i7 index primers were incorporated into library DNA via 12 PCR cycles. Libraries were purified, quantified and then pooled; denatured library DNAs were sequenced using an Illumina^®^ NextSeq^TM^ 500 sequencer using the NextSeq^TM^ 500/550 Mid Output Kit (Illumina, San Diego, CA, USA).

GenBank sequence records were collected in November 2020 and checked for new entries in May 2021. In total, 114 DHAV-1 (58 complete genomes and 56 VP1 genes) and 24 DHAV-3 (all complete genomes) sequences with available information on the date and place of collection were retrieved from GenBank. The time of origin of downloaded reference sequences encompassed a period from 1993 to 2020; sequences originated from Asia (China, Taiwan, Korea, Vietnam) and Africa (Egypt). Sequence data from the Hungarian DHAV-1 strains were generated during 2020.

### 2.2. Sequence Analysis

Complete coding sequences from short-read sequences were assembled with mapping to references using the Geneious Prime^®^ v.2020.2.4 package (16 September 2020; Biomatters, Auckland, New Zealand). The final consensus lengths of DHAV genomes were 7370–7386 nucleotides (95.6–96% coverage compared with reference strains KF953535, FJ496341 and KP721458). Regarding the sequencing results generated with Ion Torrent PGM system, 11,615 to 89,998 reads were mapped to the reference sequences, whereas the corresponding data for Illumina sequencing were 643,365 to 947,046 reads.

The BLASTn engine was used to determine similarities of the genomic sequences with relevant sequences in GenBank. Sequence alignments were prepared using the Geneious Prime^®^ v.2020.2.4 (16 September 2020; Biomatters, Auckland, New Zealand) and AliView (9 March 2021, Uppsala University, Sweden) software [19]. Pairwise sequence identities were calculated using MEGA6 v6.06 (April 2014) with the *p*-distance and pairwise deletion options [20].

Recombination analysis was carried out using RDP4 v4.101 software using the automated suite of algorithms with default settings [21]. The highest acceptable *p*-value was set to 0.05. Possible recombination signals were accepted if they were supported by independent methods with *p*-values of ≤10^−6^, after Bonferroni correction for multiple comparisons had been implemented in the program. The significant evidence of recombination, confirmed by phylogenetic analysis, was taken to represent strong evidence for recombination. The multiple-aligned sequences were cut into sections at the positions where the putative recombination breakpoints were identified, and phylogenetic analyses were carried out with MEGA6 v6.06 (April 2014) software, using the neighbor-joining method with a *p*-distance model and 1000 bootstrap replicates.

Selection constraints and the ratios of non-synonymous to synonymous mutations (dN/dS) were calculated for the coding sequences with the FUBAR (Fast Unconstrained Bayesian AppRoximation), FEL (Fixed Effects Likelihood) and MEME (Mixed Effects Model of Evolution) models of the Datamonkey server [22]. The positive selection results detected with FEL and MEME analyses were considered significant at *p* = 0.05, whereas a posterior probability of ≥0.95 was set for FUBAR calculations.

The temporal structure of the sequence dataset was checked with TempEst v1.5.3 (13 June 2019) software prior to evolutionary analysis, applying the Markov chain Monte Carlo method implemented in the BEAST package 1.10.4 (11 November 2018) [23,24,25]. After running multiple tests with various parameters, in the final analyses, the sequences were tested using lognormal relaxed molecular clock, constant size coalescent tree prior and 50 million iterations. The best fit substitution model was estimated with the smart model selection tool in PhyML 3.0 software [26]. The effective sample size and evolutionary rates were extracted with Tracer v1.7.1 [27]. The confidence level in each parameter estimate was given by the 95% highest posterior density (HPD) values. A maximum clade credibility tree was generated with TreeAnnotator v1.10.4 and visualized FigTree v1.4.2 (9 July 2014) [24,28].

## 3. Results and Discussion

When analyzing the newly determined near-complete genomes of Hungarian genotype DHAV-1 strains, the BLASTn engine found significant hits with Asian-origin DHAV-1 sequences with up to 99.85% identities (at 100% coverage). Short genomic fragments (derived from partial GenBank sequence records) were similar to Egyptian-origin DHAV-1 VP1 sequences with up to 99.4% identities.

**Figure 2:**
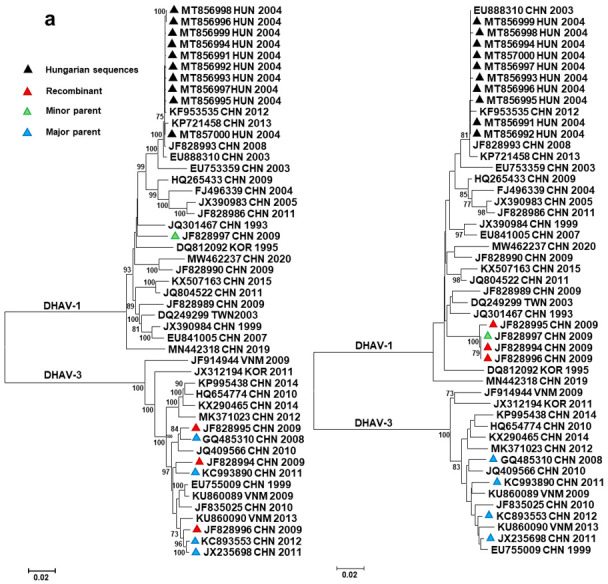

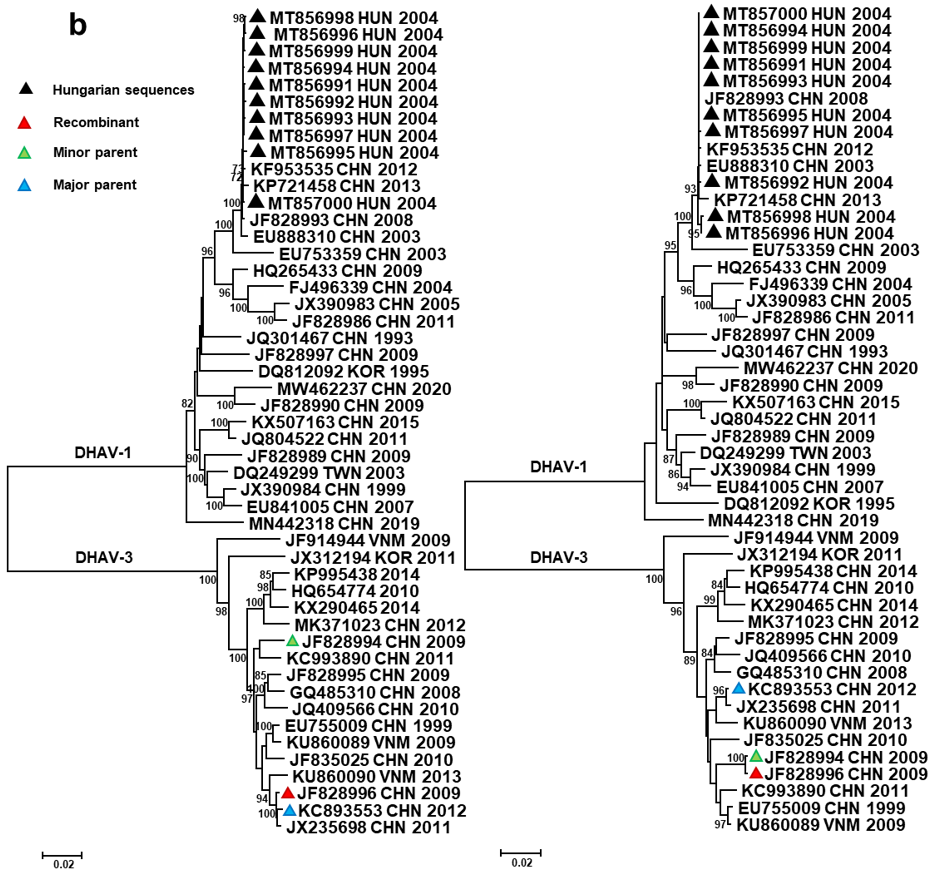
Unrooted neighbor-joining phylogenetic trees representing intergenotypic (**a**) and intragenotypic (**b**) recombination events. The trees were generated with a *p*-distance model (1000 bootstrap replicates) using sequence alignments extracted from polyprotein sequences according to the predicted recombination points determined with RDP4 software. The trees show groupings of recombinant strains with the putative major (tree on the left) and minor (tree on the right) parents (or related strains with GenBank records in all cases when true parents are unknown).

Full-length and partial genome sequences of DHAV isolates were retrieved from GenBank for further analyses. First, we investigated whether recombination affected the DHAV genomes in our data-set. Although traces of intragenotype recombination have previously been described [9], the improved methodology and the increasing number of sequence information enabled more accurate analyses to be performed. Our analyses indicated that recombination events affected the viral genomes of DHAV-1 and DHAV-3 strains, and some recombinants seemed to have acquired RNA fragments from parental strains belonging to different DHAV genotypes (Table 2, Figure 2).

In three Chinese DHAV-1/DHAV-3 intergenotype recombinants (Recombination event 1 in Table 2, Figure 2a), an ~800 nt long fragment of the 5′ genomic regions shared high sequence identity with DHAV-1 sequences (GenBank acc. no., JF828997), whereas the remaining 7 kb long genomic regions were aligned with DHAV-3 sequences (GenBank acc. no., JX235698, KC993890, GQ485310, KC893553). The recombination crossover involved the VP0 capsid-encoding region of the polyprotein in these strains. The viral protein VP0 has marked immunogenic features; thus, changes in the corresponding genomic region may contribute to immune escape mechanisms and also may influence vaccine development [5]. Of interest, although the sequence divergence within the DHAV-1 origin VP0 of Chinese intergenotype recombinants was negligible, the DHAV-3 origin genomic fragments showed marked variation, with 95.8% to 99.8% sequence identity with each other and their respective homologous sequences. This finding is consistent with the hypothesis that multiple independent DHAV-3 field strains acquired the VP0 region of a single circulating DHAV-1 parent. Of interest, an intragenotype recombination event (Recombination event 2 in Table 2, see also Figure 2b) affected a ~1300 nt long part of the 3A–3D region of the above-mentioned recombinant strain (GenBank acc. no., JF828996), in which case the major parent may have been a DHAV-3 strain very similar to a reference strain collected in China in 2012 (GenBank acc. no., KC893553), whereas the minor parent may have been another intergenotype recombinant strain (GenBank acc. no., JF828994). Strains very similar to the Hungarian strains (GenBank acc. no., MT856991 to MT857000) were also predicted to serve as putative major parents in a recombination event (Recombination event 3 in Table 2) donating a ~190 nt long fragment of the VP0 coding region to a strain collected in China in 2003 (GenBank acc. no., EU888310); in this case, the putative minor parent was similar to a strain collected in Taiwan in 2003 (GenBank acc. no., DQ249299). Although only two methods of analysis supported this event, the sequence and phylogenetic analyses (data not shown) also suggested a historic recombination event.

**Figure 3:**
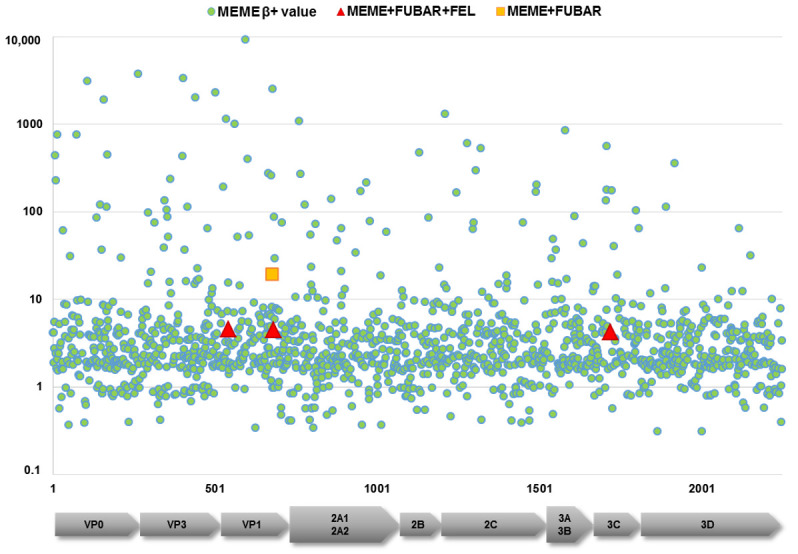
Results of the selection constraint analysis. β+ values (*y*-axis) at individual sites (*x*-axis) were extracted and plotted from results of the MEME analysis (green circles). Sites under positive selection predicted with high significance values are labeled with red triangles and the yellow square.

Next, we omitted all putative recombinants whose inclusion could have affected subsequent analyses. In the investigation of Darwinian evolution, we focused on DHAV-1, where all Hungarian strains belonged. Selection constraints were calculated for the DHAV-1 coding sequences with various models. The overall dN/dS ratio was low (≤0.159) for the polyprotein region and single genes. Positive selection was predicted to act at some aa sites, primarily in the capsid-coding VP0, VP3 and VP1 regions (Figure 3). Applying strict statistical settings, four sites were highlighted in the coding region as potential sites under positive selection constraints, including aa542 (S49PLT in VP1), aa676 (Q183RGHY in VP1), aa680 (D187VG in VP1) and aa1719 (Q104HR in 3C) of the polyprotein (Figure 3). The aa sites 183 and 187 belong to the hypervariable region of the VP1; aa substitutions of this region were connected to changes in virulence. Furthermore, changes at other sites of the VP1, as well as the VP0 and VP3 regions, may affect the virulence or facilitate immune escape mechanisms [1,29,30,31].

Evolutionary rates were estimated for each DHAV-1 genomic region coding for a mature protein. The rates for single genes were in the range of 5.6286 × 10^−4^ to 1.1147 × 10^−3^ (Table 3); these values were slightly lower than those seen in a number of other viruses within the *Picornavirales* order [32,33]. In these analyses, the polymerase-encoding 3D region showed the lowest values. In a previous study, Ma et al. [30] estimated a substitution rate of 5.57 × 10^−4^ (95% HPD 2.66 × 10^−4^ to 8.45 × 10^−4^) for DHAV-1 VP1 sequences. Analysis of an extended sample set of DHAV-1 sequences (including complete and partial genome sequences), described from strains collected between 1993 and 2020 showed a mean value of 1.013 × 10^−3^ (95% HPD 6.7481 × 10^−4^ to 1.3458 × 10^−3^) for this genomic region, which was still lower than the average rates calculated for other picornaviruses [32,33].

**Figure 4:**
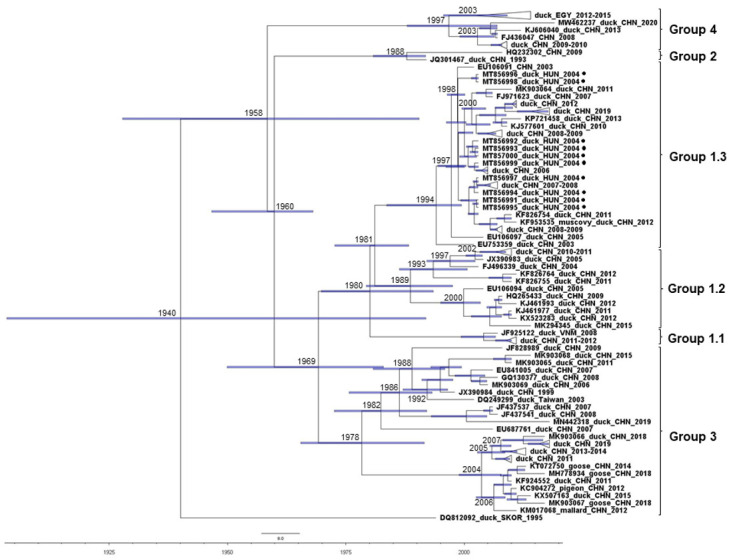
Time-calibrated maximum clade credibility tree of 102 VP1 sequences generated by the BEAST package 1.10.4, HKY + G model. Genetic groups and subgroups are on the right.

In time-scaled phylogenetic analyses of DHAV-1 sequences, some bias in sample selection was seen, given that sequences were available for strains collected over a 24-year period (and only three sequences were from before 2000) and represented only three major geographic areas: Far East Asia, North Africa and Central Europe (Figure 4). Nonetheless, the analyses showed that China may be a hotspot for the diversification of DHAV-1, with at least 60 years of evolution of the major genetic groups (GG) and subgroups (SG) of DHAV-1 [34]. Analyzing sequences of the Hungarian DHAV-1 strains, the only representative sequences from Europe, suggested that DHAV-1 was introduced on at least four occasions during the 2004–2005 Hungarian outbreak; their common ancestors had an estimated TMRCA in the late 1990s and shared an origin with multiple minor Chinese clusters whose representative strains circulated in the early 2000s and 2010s. All these strains belonged to SG 1.3; the viral genomic sequences within SG 1.3 shared 95.8–99.9% identities. The first group of Hungarian sequences (MT856996 and MT856998) showed a maximum of 99.4% identity with the closest-related reference sequences, whereas sequence identities were 99.2% (MT856991, MT856994, MT856995, and MT856997) and 99% (MT856992, MT856993, MT856999 and MT857000) for other groups of Hungarian isolates. Additionally, sequences collected from heterologous hosts clustered in a common branch (within GG 3) in the time-calibrated phylogenetic tree, a cluster which is estimated to have separated from other GG 3 strains in the late 1970s. This cluster mainly contains sequences collected after 2010 and, referring to the host spectrum, might more readily jump from the common host to a heterologous host. Another genetic group, GG 4, evolved independently from GG 1 to GG 3 strains, and contains now multiple strains from Egypt and China.

## 4. Conclusions

Evolutionary mechanisms of DHAV are similar to other picornaviruses; the main features shared by these viruses include high evolutionary rates and the potential for viral RNA recombination, even among heterotypic strains [32,33,35]. The evolutionary history and the supporting epidemiological data suggest that Asia may act as a diversification hotspot for DHAVs. The European DHAV-1 strains (represented by the genomic sequences of Hungarian isolates) are closely related to DHAV-1 strains collected in China. This is consistent with earlier observations on other closely related waterfowl origin pathogens whose geographical distribution is shared between these distant geographic areas; all these findings may suggest common drivers or modes of dispersal of a variety of waterfowl viruses between Far East Asia and parts of Europe [36,37,38]. However, currently available data are scant, which prevents detailed analyses on the route of strain dispersal between Asia and Europe; therefore, further data are greatly needed. Long-distance migrating wild birds along the circumpolar migratory route could mediate the spread of some viruses, including DHAV, known to infect waterfowls [36], although it is unknown whether any of the potential hosts susceptible to DHAV use this migratory route. The trade of domestic ducks and some ornamental bird species may also be a risk for geographical dispersal. Among heterologous hosts susceptible to DHAV, one needs to mention some columbiform and additional anseriform birds (including geese and Muscovy ducks) [1,18,39,40]. On the other hand, the routine vaccination of exported domestic breeding ducks and ducklings against DHAV-1 probably results in a reduced risk for virus spread. Concerning other DHAV serotypes, DHAV-2 and, until more recently, DHAV-3, were known to occur only in Asia. The emergence of DHAV-3 in Egypt is, however, an alarming perception. In this context, it must be kept in mind that DHAV is prone to intergenotypic recombination, and the timely detection of such recombinants is paramount for disease monitoring. In future surveillance of DHAV infections, genomic epidemiology will serve as a useful tool to track the emergence of recombinant strains with altered phenotypic features and the spread of novel non-indigenous DHAV genotypes.

## Figures and Tables

**Table 1:** Primer sequences used for the reverse transcription and amplification of duck hepatitis A virus genomes in this study.

Primer Name	Orientation	5′–3′ Sequence
DHAV_F	forward	GGAGGTGGTGCTGAAA
DHAV_R1	reverse	GTGGAAAGAAGAGAGCTGGC
DHAV_F2	forward	GATGTGGCATGTTGTYAAYCGAC
DHAV_R2	reverse	CAACCAAAYGCTGATGTTTTGG
DHAV_F3	forward	CCAACYCTTCARTGGCTCCAG
DHAV_R3	reverse	CATTTGGCTTAGGGTCCTCAC

**Table 2:** Inter- and intragenotype recombination events affecting DHAV genomes, as revealed by recombination analysis.

Event *	Region	Recombinant	Major Parent	Minor Parent	Recombination Type	*p*-Values
#1	VP0	JF828994-996 **	**JX235698, KC993890, GQ485310**	JF828997	Intergenotype	10^−11^–10^−176^
#2	3A-3D	**JF828996**	**KC893553**	**JF828994**	Intragenotype	10^−8^–10^−16^
#3	VP0	EU888310	MT856991 to MT857000	DQ249299	Intragenotype	10^−6^–10^−7^

* Three recombination events were identified. These events were arbitrarily assigned. ** Assignment of genotypes: normal letter, DHAV-1 sequence; boldface, DHAV-3 sequence.

**Table 3:** The dN/dS ratios and evolutionary rates (substitutions/site/year) estimated for individual genes of DHAV-1.

Region	Model	dN/dS	Evolutionary Rate s/s/y	95% HPD
VP0	HKY + G	0.0945	7.0037 × 10^−4^	2.3584 × 10^−4^ to 1.2087 × 10^−3^
VP1	HKY + G	0.159	7.8508 × 10^−4^	3.1147 × 10^−4^ to 1.2961 × 10^−3^
VP3	HKY + G	0.105	9.0959 × 10^−4^	3.6255 × 10^−4^ to 1.5174 × 10^−3^
2A1	HKY	0.087	1.1147 × 10^−3^	2.5081 × 10^−4^ to 2.2143 × 10^−3^
2A2	HKY + G	0.111	8.3308 × 10^−4^	3.0703 × 10^−4^ to 1.3598 × 10^−3^
2B	HKY + G	0.0489	8.112 × 10^−4^	2.2148 × 10^−4^ to 1.4842 × 10^−3^
2C	HKY + G	0.0472	6.1681 × 10^−4^	2.0083 × 10^−4^ to 1.0726 × 10^−3^
3A	GTR + G	0.0545	8.1151 × 10^−4^	2.1100 × 10^−4^ to 1.5012 × 10^−3^
3B	HKY + G	0.127	1.059 × 10^−3^	2.5708 × 10^−4^ to 1.9493 × 10^−3^
3C	HKY + G	0.0466	6.8276 × 10^−4^	2.7878 × 10^−4^ to 1.1399 × 10^−3^
3D	HKY + G	0.0719	5.6286 × 10^−4^	1.8270 × 10^−4^ to 9.9198 × 10^−4^
VP1 extended sequence set	HKY + G + I	0.191	1.013 × 10^−3^	6.7481 × 10^−4^ to 1.3458 × 10^−3^

## Data Availability

The GenBank accession number of sequences characterized in this study are MT856991–MT857000.
